# Hepatic Dyspepsia

**Published:** 1884-03-20

**Authors:** Thomas H. Line

**Affiliations:** Marquette, Neb.


### HEPATIC DYSPEPSIA.

By Thomas H. Line, M. D., Marquette, Neb.

This is an affection of which you have patients of all ages, both old and young. They complain of not being able to sleep well at night, and on arising in the morning they complain of pain in the temples and a burning sensation in the stomach. These symptoms go on increasing gradually until they begin to feel sick at the stomach at a time varying from half an hour to an hour after eating, and with this feeling there will be regurgitation of sour matter into the mouth and, very often, vomiting; sometimes, however, they cannot vomit, but there is a constant nausea and retching. There will be, in connection with these symptoms, a peculiar sensation in the bowels. They will tell you that it acts as though something was biting them, and occasionally ask if it is not worms working in their bowels, or "running around" as they say. This sensation occurs about the navel and is accompanied by frequent flashes of heat and cold, alternately; occasionally a chill occurs.

We have now brought forward symptoms which point to several distinct functional disturbances, all of which belong primarily to a functional disturbance of the liver, and latterly those belonging to a functional disturbance of the stomach. Most of the symptoms can, however, be referred to a disorder of the liver. It is probable that this derangement of the liver is due to the fact that not a sufficient quantity of bile is furuished for purposes for which it is needed. This may be due to the liver's inability to produce enough bile, or it may be due to the obstruction of the gall ducts, thus preventing its being eliminated. I am inclined to believe that the latter is the cause of the cases that came under my observation. Hard water or strong lime water seem to be predisposing causes. To relieve these symptoms is a question that will sometimes trouble the best of us. As a rule, they have been taking all kinds of patent medicines, and only when they are unable to stand it any longer they apply to you for treatment. Some will tell you to put them on iron, quinia or some other tonic. I think a treatment of this kind will almost invariably fail. The treatment should be directed mainly to the liver, which is the cause of the symptoms in other parts of the body, and so, in the first place, I put them on such a treatment as follows:

℞ Podophylin........................ one-fifth grain,
Hydrarg bichlorid............... one-tenth grain,
Ext. colocynth comp............. three-quarter grain.
Ft. Capsule No. 1.

I give one of these capsules in the above proportion three times a day, and gradually lessen the number as the symptoms improve. The diet should be composed principally of animal broths and meat. Then it is well to advise them to wash themselves all over every morning with cold water and rub dry with a rough towel. This, with fresh out-door exercise, will generally bring them out all right.—*Peoria Med. Monthly.*

---

**Tarnished** colored gold articles, it is said, may be restored by the following method: Dissolve one ounce of bicarbonate of soda, half an ounce of chloride of lime and half an ounce of common salt in about four ounces of boiling water; take a clean brush and wash the article with the hot solution for a few seconds, and rinse them immediately in two clean waters; after this process, dry them in warm sawdust, and finish the operation by rubbing them over with tissue-paper.

